# “What You Need, Baby, I Got It”: Transposable Elements as Suppliers of Cis-Operating Sequences in Drosophila

**DOI:** 10.3390/biology9020025

**Published:** 2020-02-03

**Authors:** Roberta Moschetti, Antonio Palazzo, Patrizio Lorusso, Luigi Viggiano, René Massimiliano Marsano

**Affiliations:** 1 Dipartimento di Biologia, Università degli Studi di Bari “Aldo Moro”, Via Orabona 4, 70125 Bari, Italy; roberta.moschetti@uniba.it (R.M.); lorusso.patrizio@libero.it (P.L.); luigi.viggiano@uniba.it (L.V.); 2 Laboratory of Translational Nanotechnology, “Istituto Tumori Giovanni Paolo II” I.R.C.C.S, Viale Orazio Flacco 65, 70125 Bari, Italy; a.palazzo@oncologico.bari.it

**Keywords:** transposable elements, *Drosophila melanogaster*, cis-regulatory elements, promoter, enhancer, insulator, heterochromatin, genome evolution

## Abstract

Transposable elements (TEs) are constitutive components of both eukaryotic and prokaryotic genomes. The role of TEs in the evolution of genes and genomes has been widely assessed over the past years in a variety of model and non-model organisms. *Drosophila* is undoubtedly among the most powerful model organisms used for the purpose of studying the role of transposons and their effects on the stability and evolution of genes and genomes. Besides their most intuitive role as insertional mutagens, TEs can modify the transcriptional pattern of host genes by juxtaposing new *cis*-regulatory sequences. A key element of TE biology is that they carry transcriptional control elements that fine-tune the transcription of their own genes, but that can also perturb the transcriptional activity of neighboring host genes. From this perspective, the transposition-mediated modulation of gene expression is an important issue for the short-term adaptation of physiological functions to the environmental changes, and for long-term evolutionary changes. Here, we review the current literature concerning the regulatory and structural elements operating in *cis* provided by TEs in *Drosophila*. Furthermore, we highlight that, besides their influence on both TEs and host genes expression, they can affect the chromatin structure and epigenetic status as well as both the chromosome’s structure and stability. It emerges that *Drosophila* is a good model organism to study the effect of TE-linked regulatory sequences, and it could help future studies on TE–host interactions in any complex eukaryotic genome.

## 1. Introduction

Transposable elements (TEs), also known as “jumping genes”, are exceptional modifiers of the genome structure and gene expression. Since their discovery and characterization in eukaryotic genomes in the 1940s [1], TEs have long been regarded as junk DNA, useless and harmful sequences that replicate in the genome with no advantage conferred to the host [2,3]. Nowadays, there is a considerable amount of evidence against the junk DNA hypothesis. With few known exceptions, eukaryotic genomes are densely wrapped with TEs [4] that contribute to their adaptation and evolution [5].

TEs move around in the host genome using self-encoded enzymes that catalyze the transposition reaction. Members of Class I adopt a replicative transposition, directly resulting in an effective increase of the TE copies per genome after the completion of the transposition, while Class II elements perform a conservative transposition, i.e., yield an identical copy number after the transposition. 

Members of Class I (retrotransposable elements, RTE) transpose via reverse transcription of an RNA intermediate that is afterward integrated into a new genomic locus. The two main subclasses are represented by the LTR-containing retrotransposons and the non-LTR retrotransposons. Besides their main structural difference (i.e. the presence of LTR, long terminal repeats, terminal directly repeated sequences), they are extremely different in their mechanism of transposition. LTR-retrotransposons perform transposition in a way comparable to that of retroviruses, priming the reverse transcription process with the 3′ end of an endogenous tRNA molecule and two distinct template jumps that allow the completion of the cDNA synthesis [6]. The main enzymatic activities that take part in the replication process (reverse transcriptase and RNAseH), are RTE-encoded. The integrase enzymatic activity completes the retrotransposition with the integration of the new copy. The retrotransposition of non-LTR retrotransposons also relies on the reverse transcriptase activity that in this case is primed by a free 3′ single-strand DNA end at the cleaved insertion site, a mechanism known as target primed reverse transcription (TPRT) [7].

TEs belonging to Class II are also called DNA transposons. Usually, they contain terminal inverted repeats (TIRs), flanking the transposase gene that encodes an integrase essential to perform the transposition step, known as the cut-and-paste mechanism. The transposase excises the donor transposon and inserts it into a new locus through a TE-specific recognition of the TIRs.

For many years, *Drosophila melanogaster* has been considered as a warhorse for genetic studies. Indeed, the fly cultures’ low management costs together with its short life-cycle, the support earned from more than a century-long story in genetics studies, and the availability of sophisticated toolkits and protocols for genetic investigations [8,9] have strongly consolidated this model organism, making it unparalleled compared to other animal models. Also, the genome of *D. melanogaster* was one of the earliest sequenced animal genome [10], even in its heterochromatic compartment [11,12]. *D. melanogaster* is currently widely used as an animal model to study the most diverse aspects of genetics, from basic inheritance to cancer [9], but additional genomic resources are continuously developed for other species of the *Drosophila* genus that will soon become model species in specific fields of investigation [13,14,15,16,17].

## 2. Drosophila TEs: A Brief Overview

The earliest hypothesis on the presence of TEs in the genome of *D. melanogaster* date back to the late 70s, with the observation that repetitive sequences inserted at new sites in vitro [18] and in vivo [19]. During the same years, the “P factor hypothesis” [20]-a transposon-linked explanation of the *Drosophila* P-M hybrid dysgenesis-was confirmed [21,22]. Shortly after, in the early 1980s, the instability of an eye-color phenotype was associated with the presence of extra DNA inserted in the proximity of the white locus [19,23,24].

After that, many known repetitive sequences proved to be TEs. The molecular characterization of some of them led in the following years to the development of powerful insertional mutagenesis tools such as *P-element* from *D. melanogaster* [25,26] and *Minos* from *D. hydei* [27].

**Figure 1:**
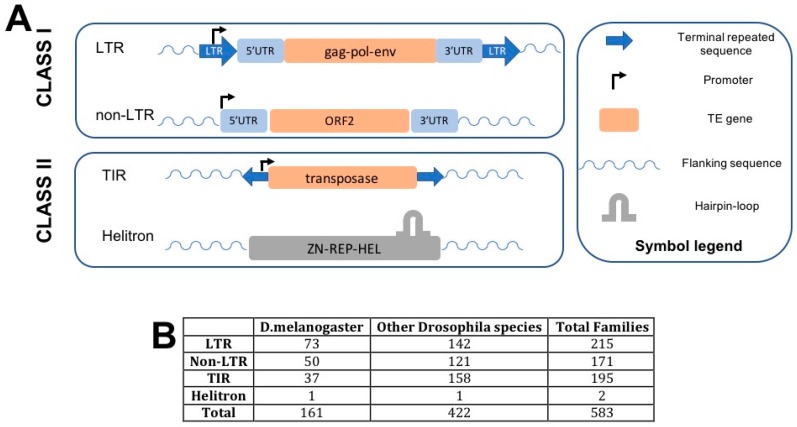
(**A**) Structural features of the TEs identified and described in *Drosophila*. The symbols used are described on the left part of the panel. (**B**) Overview of the number of TE families belonging to each of the main groups of TEs in *D. melanogaster* and in other *Drosophila* species (source: http://flybase.org, last access December 2019).

The *D. melanogaster* genome sequence draft offers the opportunity to annotate a reference mobilome [28]. Afterwards, few additional transposon families, mainly residing in the heterochromatin or absent in the reference strain, were discovered and characterized [29,30,31]. This set of information has been complemented with the genome sequencing of 69 additional species of the *Drosophila* genus https://www.ncbi.nlm.nih.gov/genome/?term = drosophila-last (accessed on 24 December 2019) and the TE characterization in non-model *Drosophila* species, leading to the possibility to perform large comparative and evolutionary studies [32,33,34,35]. Figure 1A summarizes the main structural features of the TE types in the genome. The number of families currently annotated in the genome of *D. melanogaster* as well as in other *Drosophila* species is reported in Figure 1B. 

TEs of both classes occupy roughly 20% of the genome of *D. melanogaster*. It has been estimated that nearly 30% of the TE complement (20% of the DNA transposons, 21% of non-LTR retrotransposons and 45% of LTR retrotransposons respectively) in *D. melanogaster* consists of full length and potentially active elements [28]. 

Usually, all TE families consist of a non-autonomous element in addition to transposition-competent elements. At least a fraction of non-autonomous elements, that usually exceed in number the autonomous one, could be still mobilized by the in *trans* action of the wild type transposition machinery expressed from autonomous elements. It is believed that trans-mobilized non-autonomous elements are the principal contributors of the dissemination of *cis*-acting regulatory sequences throughout the genome, inducing transcriptional network rewiring and the alteration of wild type transcriptional patterns [36].

Few Helitron families are also annotated in the reference genomes of sequenced Drosophila species. *Helitrons* encode a 5′-to-3′ DNA helicase and nuclease/ligase similar to those encoded by rolling-circle replicons, and process a single stranded DNA intermediate that replicate using the rolling-circle replication mechanism.

It is remarkable that no active DNA transposons have been identified in humans and mice [37] nor in the vast majority of mammals, with the exception of some bat species [38,39,40,41], thus limiting the possibility to investigate in these species the short-term effect of insertions mediated by this group of TEs. *D. melanogaster* as well as other *Drosophila* species, are therefore promising model organisms for studying the contribution to regulatory sequences by eukaryotic TEs. 

Here, we will review the current knowledge on the *cis*-acting sequences identified in TEs and their effects on gene expression and genome architecture. Their contribution is indeed not limited to sequences that affect (either positively or negatively) the transcription of genes, but extends to sequences with important structural functions given their ability to recruit chromatin proteins. A list of known TE-insertions contributing *cis*-acting sequences in *Drosophila* is reported in Table 1. 

**Figure 2:**
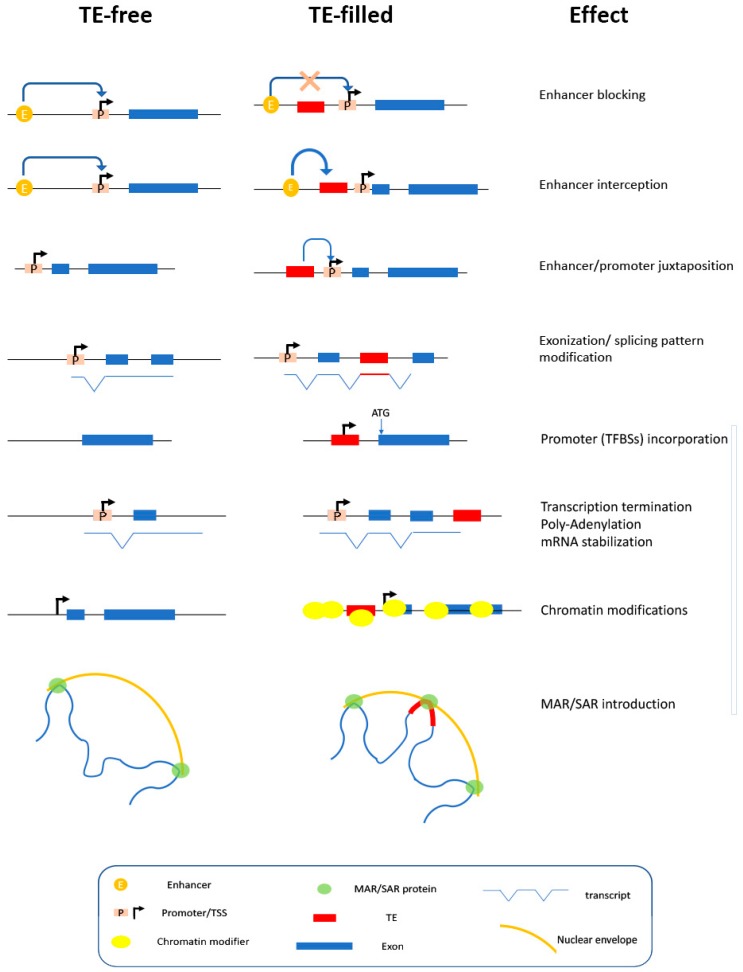
Schematic representation of the key effects produced by the *cis*-operating sequences upon TE insertion. Symbols are explained in the box.

The contribution in *cis*-acting sequences provided by TEs is described in below and is summarized in Figure 2.

## 3. TEs as Promoter Suppliers

The promoter region is defined as a *cis*-regulatory sequence that assembles the pre-initiation complex (PIC) [80] to recruit the RNA polymerase, which starts the transcription process. Promoters are modular sequences containing transcription factor (TF) binding sites (TFBSs), consisting of short sub-sequences that are recognized, more or less specifically, by TFs. Just like non-mobile genes, TEs need promoters to start transcription. TE-associated promoters are recognized by the same RNA polymerases that operate in the nucleus and thus must contain species-specific promoter motifs in order to assemble the PIC and start transcription. The exceptional mobile ability of these sequences allows the incorporation of new TFBSs in the proximity of promoter-less coding sequences or their juxtaposition to existing promoters. In the first case, new transcripts can be generated from previously non-expressed sequences, such as retroposed pseudogenes, leading to the birth of new genes, a relevant event in the evolution of genomes.

Many cases of transposition-mediated promoter acquisition have been described in *Drosophila* (Table 1). Elements belonging to both classes of TEs can provide promoter sequences to resident genes, thus originating relevant phenotypes. Besides specific studies demonstrating that individual TE insertions modify the expression of nearby genes, a systematic study by Batut and colleagues suggested that TEs contribute large number of developmentally expressed transcriptional start sites and can distribute pre-assembled *cis*-regulatory modules in the genome [64].

Furthermore, the promoters of elements belonging to the *Bari* family [34] have been recently tested for their ability to drive a reporter gene expression in expression vectors [81,82]. While the promoter of LTR retrotransposons such as *copia*, *ZAM* and *Tirant* strongly supported the reporter transcription, the promoter of two DNA transposons, *Bari1* and *Bari3*, turned out to be weak promoters [81]. Surprisingly, the promoters of the *Bari* transposons show an inter-Domain transcriptional activation [81], which is not displayed by other elements, suggesting that they evolved special features enabling their spread in other genomes. Interestingly, this feature seems to be conserved among the members of the *Tc1/mariner* superfamily [82].

It has been also shown that many retrotransposons and a few TIR elements are transcribed bi-directionally, starting from internal canonical RNA polymerase II promoters, an observation that deals with their regulation through the RNA interference pathway both in somatic and in germline tissues [83,84].

A recent study performed using bioinformatic prediction coupled with Chip-seq data has revealed a significant enrichment of stress-related TFBSs in TEs [85], definitively supporting the idea that TEs are involved in stress responses.

TEs turned out to be an important source of promoters also in the heterochromatin. Heterochromatic genes are regularly transcribed in *Drosophila* [86] and their promoters have peculiar structural and functional features compared to euchromatic gene promoters [87]. As proposed by Yasuhara et al. [87] “an attractive possibility is an acquisition of TE-derived promoters given the predominance of TE-like sequences in heterochromatin and the finding that some TE promoters are transcribed in heterochromatin”.

### 3.1. Enhancers, Silencers and Insulators within TEs

Repeated DNA in the form of a simple or complex minisatellite has been frequently observed in the UTRs of many retrotransposons. This apparently unusual feature has been associated with the ability to form complexes with DNA binding proteins, thus interfering with the transcription of nearby genes by modifying the chromatin status of the locus. Indeed, the tandem repeat DNA within the UTRs of retrotransposons contains arrays of protein binding sites that are associated with either enhancer or insulator functions. An analysis of the UTRs of retrotransposons performed by Minervini and colleagues [50] provided evidence that repeats are commonly found in the *Drosophila* RTEs.

The first well-characterized function associated with a tandem repeat within a TE was found in the 5′UTR of the *copia* retrotransposon [88]. Later on, a potent insulator was characterized in the 5′UTR of the *gypsy* retrotransposon [89]. This is a 350 bp-long sequence consisting of an array of 12 degenerated binding sites for the su(Hw) gene product, a DNA binding protein that determines the insulator function. The potency of the *gypsy* insulator depends on the amount of su(Hw) binding sites [90]. Another efficient insulator has been characterized in the LTR of *Idefix* [48].

Repeated DNA sequences within the 5′UTR of some retrotransposons may also act as transcriptional enhancers. The first well-characterized retrotransposon-associated enhancer in *D. melanogaster* is *ZAM* [48]. *ZAM* was formerly discovered in a fly strain displaying an unstable eye-color phenotype over time [31].

The LTR-retrotransposon *Accord* provides an additional example of retrotransposon-associated enhancer. Indeed, fly populations carrying an *Accord* insertion upstream the *cyp6g1* gene are resistant to DDT [91] and nicotine [92] due to the augmented expression of the *cyp6g1* gene.

Silencers associated with TEs are poorly described in the scientific literature. However, a silencer has been recently identified and characterized in the *D. melanogaster Mos1* element, which belongs to the *Tc1/mariner* superfamily [93]. This was a bit surprising given the simple and compact structure of the *mariner*-like elements, which is expected to contain minimal *cis*-regulatory sequences (e.g., promoters). Also surprising is the evolutionary conservation of silencers in a homologous region of other animal *mariner*-like elements suggesting that either the silencer function is very ancient, or it might have been raised several times in the *mariner* elements during animal evolution [93]. Interestingly, the *gypsy* insulator behaves as a silencer depending on the genetic background [62], a situation that clearly shows the versatility of some TE-linked regulatory sequences. A similar duality has been also highlighted for the *ZAM* 5′UTR, which behaves as an enhancer when tested in vivo [48] while it acts as an insulator when tested in cultured cells [94].

### 3.2. Additional Cis-Regulatory Transcriptional Signals within TE

In addition to the above-described functions, TEs are also a source of *cis*-acting sequences involved in the transcription termination, splicing, and mRNA stability. TE insertions within genes could alter the splicing pattern of primary RNAs depending on the strength of the splicing consensus introduced upon insertion, further increasing the transcriptome variability of the host genome.

POGON1 and *Bari1* supply poly-adenylation signals that increase the expression of the gene located upstream their insertion sites, conferring a relevant xenobiotic-resistance phenotype to population bearing such insertions [76] [72]. A transcription termination site has been also described in the 5′UTR of *MDG1* element [68].

TEs also provide splicing sites. TEs can modify the exon/intron structure with the introduction of splicing consensus sequences, allowing the incorporation of TE sequence into the mRNA. This phenomenon, called TE exonization [95], has been recently observed in the brain of *D. melanogaster* in which newly inserted copies of TEs are expressed in a way directly correlates with that of neighboring genes [96]. 

While splicing is a common post-transcriptional modification in retrotransposons, it is less frequent in members of Class II TEs. *P-element* is a DNA transposon of *D. melanogaster* that possess introns that are spliced out with a tissue-specific pattern [97]. Interestingly, spliced RNA isoforms have been described in two active *Tc1/mariner* elements. While these elements contain intron-less transposase gene, their transcripts are spliced following the canonical (*Bari3* [98]) or the unconventional (*Bari1* [99]) splicing when over-expressed in experimental model systems.

### 3.3. Structural Role of Cis-Operating Sequences within TEs

Besides the transcriptional control elements, TEs contain *cis*-acting sequences that might influence the epigenetic status of the insertion locus. It has been experimentally demonstrated that arrays of three or more *P-elements* carrying a *white* reporter gene produce a variegated eye phenotype [100] similar to the classical heterochromatin-induced position-effect variegation [101]. This was the first experimental demonstration of the ability of TEs to seed heterochromatin in virtually every genomic site. Similar behavior was observed for the *1360* transposon and for the *invader4* retrotransposon [77] suggesting a broad ability of TEs to induce heterochromatin formation. This ability is granted by the recruitment of heterochromatic proteins such as HP1 at the site of insertion [77]. HP1a, and to some extent HP1b, are key heterochromatin-associated proteins that can interact with a plethora of additional chromatin proteins [102] that can mediate the establishment of repressive chromatin marks. HP1 binding ability has been observed for several TE families [103] [50].

The ability of TEs to introduce new chromatin protein binding sites upon insertion is also relevant in the context of the rewiring of pre-existing transcriptional circuits. An amazing example is the evolutionarily new X chromosome in *D. miranda* that has accumulated hundreds of MSL complex (male-specific lethal complex) binding sites provided by reiterated insertion of *ISY* [104], a *Helitron* element. The MSL complex is recruited to high-affinity chromatin entry sites on the *Drosophila* male X chromosome and spreads in *cis* to coordinate the expression of X-linked genes, thus achieving dosage compensation. In *D. miranda*, the accumulation of *ISY* has led to switching off the dosage compensation system on the old X chromosome, rewiring it to the newly emerged (neo-X) sex chromosome [71].

In this context, the role of TEs in maintaining the centromeres and the telomeres in *Drosophila* is well known. A profound cooperation between three *LINE*-like elements (*HeT-A*, *TAHRE*, and *TART*) allows both their transposition and stability of host chromosomes [105]. In addition, a suggestive hypothesis has been proposed that directly links the organization and function of centromeres of *D. melanogaster* and *D. simulans* to the ability of the *G2/Jockey-3* transposon to recruit the centromeric protein CENP-A [106]. 

piRNA clusters (or piRNA loci) are TE-dense heterochromatic loci from which piRNAs are produced to defend the host genome from transposition in the germline [107]. It has been demonstrated that sequences sharing homology to piRNAs operate as *cis*-acting targets for heterochromatin assembly, which is usually associated with HP1a and H3K9me2/3 [77]. In this context, many TEs can aid in establishing the epigenetic organization of the piRNA loci in *Drosophila* as well as in other organisms.

Notably, the same *gypsy* sub-sequence that contains the insulator/silencer function (described above), also functions as MAR/SAR (matrix attachment region/scaffold attachment region), connecting a transcriptional role to a structural role of the *gypsy* retrotransposon [69]. A sequence displaying MAR function was also identified and characterized in the *roo* element [70]. Although this aspect is not deeply investigated, these results highlight a *cis*-structural role of TEs, whose importance is comparable to the role of TEs in centromeres and telomeres.

## 4. Conclusions and Future Directions: What Can We Still Learn from *Drosophila*?

Many phenotypes that have been partially characterized in *D. melanogaster* might be due to the introduction of new *cis*-regulatory elements resident in transposons.

**Figure 3:**
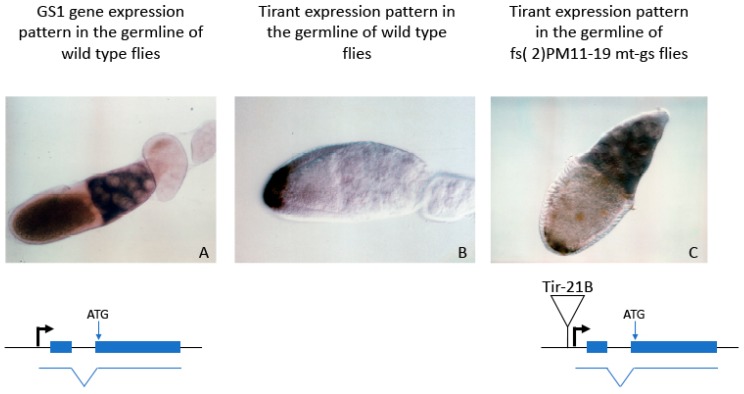
In situ hybridization performed on wild type (panels **A** and **B**) or mutant (panel **C**) ovaries using *GS1* (panel **A**) or *Tirant* (panels **B** and **C**) specific probes. The organization of the relevant locus in the 21B region of the polytene chromosomes of *D. melanogaster* is provided.

As an example, there is evidence suggesting that *Tirant*, an LTR-retrotransposon, could also carry an insulator. The insertion of a defective copy of *Tirant* in the 21B region, upstream of the *GS1* gene (fs(2)PM11-19 mt-gs), has been previously reported to cause a hypomorphic mutation that raises a female-sterile phenotype [49]. Notably, upon insertion, the *Tirant*-21B element acquired a transcriptional pattern that is the perfect merge of the *GS1* and the wild type *Tirant* patterns (Figure 3). 

This situation is compatible with the presence of an insulator function within *Tirant* that in turn focuses the *GS1* enhancer action on its own promoter. From an evolutionary point of view, this could be a strategy that increases the expression of RTE-related genes in specific tissues, such as the germline.

Little is known about the role of TE insertions into the Y chromosome. This entirely heterochromatic chromosome, while dispensable for male fly viability, is essential for male fertility, since it carries genes that are important in spermatogenesis. These are among the largest genes known in *D. melanogaster* and their transcription mechanism has been recently disclosed [108]. If possible, less is known about the *cis*-effect exerted by transposon islands that populate the centromeric DNA of *D. melanogaster* chromosomes. The organization of the centromeric DNA has been determined using mini-chromosomes obtained by progressive deletions, which confirmed previous data on the satellite and transposon islands populating the centromeric DNA in *Drosophila*. TEs are responsible for neuronal mosaicism in the mushroom bodies of *D. melanogaster* [109,110]. A similar transposition-based genetic mosaicism was described in the hippocampal neurons of the human brain [111,112], suggesting a conserved role of TEs as the basis of the genetic and functional diversification in the cells of particular neuronal districts in the animal’s brain. Additional effort will be necessary to fully understand how TEs modify the transcriptional profile at the single neuron level and the impact at a larger scale neurological level.

TEs densely populate the centromeric and pericentromeric heterochromatin of *D. melanogaster*. Their arrangement, in combination with simple and complex satellites, is a feature of the centromeric DNA whose importance is still undeciphered relative to centromere function. An interesting aspect of the presence of TEs in the pericentric heterochromatin is the presence of TE clusters. One of them has a peculiar feature. The *Bari1* cluster maps in the h39 region of the second chromosome of *D. melanogaster*, adjacent to the XbaI repeat that identify the Responder (*Rsp*) locus [34]. While apparently devoid of functional significance, this region has been proven to be important for some fitness-related performance of the species [113]. However, while the *Rsp* cluster is highly polymorphic, the *Bari1* cluster shows high structural conservation, in terms of copy/number, in many populations tested so far [114]. This could be the result of an unexplored *cis*-effect on the centromere or on the whole chromosome. In vitro and in vivo studies using DNA adenine methyltransferase identified HP1 binding sites within the *Bari1* cluster [75], reinforcing its structural role in the establishment of the heterochromatin domain in the h39 region. New methodological approaches, such as genome editing [115], could enable the discovery of new functions associated to heterochromatic TE-clusters.

## Figures and Tables

**Table 1:** List of reported *cis*-regulatory elements provided by TEs in *Drosophila* species. Species are indicated with a four-letter code (the first letter specifies the Genus, the following three specify the species).

Species	Affected Gene/Locus	TE	Transposon Type	*Cis*-Regulatory Activity	Effect	Evidences	Reference
Dmel	Cyp6g1	Accord	LTR	Enhancer	Increased xenobiotic resistance	Reporter Assay	[42]
Dsim	Cyp6g1	*Doc*	non-LTR	Enhancer(?)	Increased xenobiotic resistance	DDT resistance and gene over-expression	[43]
Dmel	bxd1	gypsy	LTR	enhancer	Development of thoracic segment	Phenotype assay	[44]
Dmel	Telomeres	HeT-A	non-LTR	Telomere elongation	Telomere maintainance	In vivo assay	[45]
Dmel Dvir	Telomeres	TART	non-LTR	Telomere elongation	Telomere maintainance	In vivo assay	[46]
Dmel	Telomeres	TAHRE	non-LTR	Telomere elongation	Telomere maintainance	DNA sequencing	[47]
Dmel	white	Idefix	LTR	insulator	Eye pigmentation	Phenotype assay	[48]
Dmel	GS1	Tirant	LTR	ND	ND	Direct assay	[49]
Dmel	NA	ZAM	LTR	HP1 binding	Chromatin state determination	In vitro assay	[50]
Dmel	NA	copia	LTR	Enhancer	Reporter expression	Direct assay	[51]
Dmel	development genes	17.6, 297, 412, 1731, 3S18, blood, copia, gypsy, HMS Beagle Kermit/fleamdg1 mdg3 opus B104/roo springer	LTR	Cis-regulatory sequences	alterations of gene expression during embryogenesis	Expression analyses	[52]
Dmel	Hsp70Ba	jockey	LTR	Cis-regulatory sequences	suppression of the deleterious phenotypes of Hsp70.	Phenotypic assay, expression analysis	[53,54]
Dmel	87A7 hsp70	H.M.S. Beagle	LTR	Unknown	suppression of the deleterious phenotypes of Hsp70.	Phenotypic assay, expression analysis	[53]
Dmel	LCP-1 LCP-f2	H.M.S. Beagle	LTR	enhancer-like elements	Transcriptional activation of LCP genes	Genetic variants analyses	[55] [56]
Dmel	achaete-scute complex	*transpac*	LTR	enhancer-like elements	variation in bristle number	Genetic variants analyses	[57] [58]
Dmel	kuz	F-element	non-LTR	cis-regulatory	Gene up- regulation	Population analyses	[59]
Dmel	yellow	gypsy	LTR	insulator	Yellow phenotype	In vivo analyses	[60] [61] [62]
Dmel	NA	B104/roo	LTR	promoter	NA	Inferred from in vivo assay	[63]
Dmel	*TM4SF*	*297*	LTR	promoter	NA	RNA ligase-mediated 5′-RACE	[64]
Dmel	152 annotated genes	roo gypsy Pao	LTR	Promoter	NA	RNA ligase-mediated 5′-RACE	[64]
Dsim	Slowpoke	Shellder	LTR	Altered splicing	Courtship song variation	Trait mapping, in vivo CRISPR knockout	[65]
Dmel	CG18446	roo	LTR	alternative transcription start site	increased expression	5′-RACE	[66]
Dana	Om(10)	TOM	LTR	enhancers	Eye morphogenesis	In vivo assay	[67]
Dmel	NA	MDG1	LTR	Transcription termination	NA	Transcriptional analysis	[68]
Dmel	NA	gypsy	LTR	MAR	NA	In vivo assay	[69]
Dmel	NA	roo	LTR	MAR	NA	In vivo assay	[70]
Dmir	Neo X	ISY	Helitron	MSL binding site	Dosage compensation	Direct assay	[71]
Dmel	HSP70BA	P-element	DNA	Silencer	Reduction of Hsp70 expression level.	Direct phenotypic assay	[54] [53]
Dmel	CG11699	POGON1	TIR	Poly-A signal	Increased xenobiotic resistance	3′ RACE	[72]
Dmel	Jheh1, Jheh2	Bari1	TIR	HP1 seeding	Antioxidant response	Phenotypic assay	[73] [74]
Dmel	h39 region	Bari1	TIR	HP1 binding	Chromatin state determination	Direct assay	[75]
Dmel	Cyp12a4	Bari1	TIR	polyA signal	detoxification	3′ RACE	[76]
Dmel	NA	1360/hoppel	TIR	Hp1 recruitment	Heterochromatin formation	In vivo assay	[77]
Dsim	hunchback even-skipped	hoboVA	TIR	Promoter, transcription factor binding sites (TFBSs)	new phenotype	Expression and in situ analyses	[36,78]
Dmel	Hsp70Bb	S-element	TIR	cis-regulatory	NA	population genetics study	[79]
Dmel	rdx	S-element	TIR	cis-regulatory	down-regulation	Population analysis	[59]
Dmel	152 annotated genes	Tc1 P hAT Helitron	TIR Helitron	Promoter TSS clusters	NA	RNA ligase-mediated 5′-RACE	[64]
